# Altered anterior cingulate cortex functional connectivity in treatment-naïve obsessive-compulsive disorder: a resting-state fMRI study

**DOI:** 10.3389/fpsyt.2026.1835812

**Published:** 2026-05-26

**Authors:** Yujing Li, Lisha Ma, Jun Hu, Zonghong Li, Xiaofang Shang, Shu Xu, Junjun Liu

**Affiliations:** 1 Department of Psychiatry, Nanjing Medical University Affiliated Brain Hospital, Nanjing, China; 2 Department of Psychosomatic Medicine, Zhejiang University Affiliated Hangzhou Seventh People’s Hospital, Hangzhou, China; 3 Department of Radiology, Nanjing Medical University Affiliated Nanjing Brain Hospital, Nanjing, China; 4 Department of Psychology, Nanjing Medical University Affiliated Brain Hospital, Nanjing, China; 5 Department of Psychiatry, Nanjing Meishan Hospital, Nanjing, China

**Keywords:** Anterior cingulate cortex, functional connectivity, obsessive-compulsive disorder, resting-state fMRI, treatment-naïve

## Abstract

**Objective:**

To investigate the anterior cingulate cortex (ACC) resting-state functional connectivity patterns in OCD patients who have not yet received therapy and analyze how they relate to the intensity of their clinical symptoms.

**Methods:**

Resting-state fMRI data were acquired from 46 medication-naïve participants with OCD and 33 demographically comparable neurotypical control subjects. The region of focus for the seed-based whole-brain functional connectivity investigation was bilateral ACC. Relationships between aberrant connections and clinical characteristics measured by the Y-BOCS, HAMD-17, and HAMA were investigated using Pearson’s correlation coefficient and partial correlation analysis.

**Results:**

Key findings (OCD patients vs. healthy controls): Increased functional connectivity (FC) in OCD patients: Right insula (Brodmann area 48), Right hippocampus (BA 20), Right fusiform gyrus (BA 37); Decreased FC involving the anterior cingulate cortex (ACC) with: Right supplementary motor area (SMA, BA 32), Left inferior frontal gyrus (IFG, BA 47) (AlphaSim corrected, P < 0.05). Clinical association: There was a significant positive relationship between Y-BOCS total scores (a measure of OCD symptom severity) and FC strength linking the left IFG to the ACC (r = 0.351, P = 0.017). In practical terms, greater symptom severity is associated with stronger coupling between these two regions. No other clear brain-behavior relationships were found in the other regions examined.

**Conclusion:**

Treatment-naïve OCD patients demonstrate distinct ACC functional connectivity alterations involving cognitive control, motor planning, and limbic processing regions. The specific association between left inferior frontal gyrus (IFG)-ACC connectivity and symptom severity suggests that this pathway may serve as a neurobiological marker for OCD pathophysiology.

## 1 Introduction

About 2–3% of the worldwide population is affected by obsessive-compulsive disorder (OCD), a persistent and debilitating mental illness characterized by unwanted obsessive thoughts and recurrent compulsive behaviors or mental acts (1, 2). The disorder typically emerges in adolescence or early adulthood and imposes substantial functional impairment and a reduced quality of life on affected individuals (3). Converging evidence from neuroimaging research over the last three decades has linked a key pathophysiological characteristic of OCD to disruptions in the cortico-striato-thalamo-cortical (CSTC) circuits (4, 5). This traditional neurocircuitry model posits that an imbalance across the excitatory direct and inhibitory indirect neural circuits within the broader CSTC system underlies the characteristic loss of cognitive and behavioral control observed in OCD patients (6). The anterior cingulate cortex (ACC), one of the circuit’s major nodes, is particularly important for emotion regulation, cognitive control, error detection, and conflict monitoring (7, 8). There have been repeated reports of ACC structural and functional abnormalities in OCD, including increased regional activity during symptom provocation tasks and altered gray matter volume (9, 10). In parallel, the ACC interacts strongly with the orbitofrontal cortex (OFC), dorsolateral prefrontal cortex (DLPFC), and striatum (notably the caudate and putamen) as well as the thalamus, forming part of a larger network that includes limbic structures such as the amygdala, hippocampus, and insula (11, 12). Accumulated evidence from resting-state fMRI over recent years indicates that obsessive-compulsive disorder involves abnormalities in the CSTC circuit and widespread disruptions across large-scale networks, notably three core intrinsic brain networks — the salience network (SN), the default mode network (DMN), and the executive control network (ECN) (13, 14).According to these results, examining the ACC’s intrinsic functional connectivity patterns at rest may offer vital information about the brain processes underlying the pathophysiology of OCD and how they relate to the intensity of clinical symptoms.

Without imposing explicit externally directed cognitive requirements, rs-fMRI has established itself as a robust and widely adopted approach to characterize the human brain’s inherent neural activity and functional interconnections, offering unique advantages in identifying spontaneous neural network alterations associated with psychiatric disorders (15). Numerous large-scale brain networks have shown significant abnormalities in functional connectivity in OCD patients, according to recent rs-fMRI studies. In particular, abnormalities within the DMN—a large-scale intrinsic brain network associated with spontaneous internally oriented thought and self-related cognitive processes—have been well-documented in existing literature (13, 16);abnormalities have also been reported in the executive control network, which subserves goal-directed cognitive processes and working memory (5, 17); and in the salience network, which facilitates the detection of behaviorally relevant stimuli and coordinates switching between other networks (18). With regard to ACC-based functional connectivity, several studies have documented aberrant connectivity patterns between the ACC and areas such as the prefrontal cortex, thalamus, striatum, and limbic systems in OCD patients (7, 10, 19).For instance, decreased ACC-striatal connectivity has been associated with impaired cognitive flexibility (5), while enhanced ACC-limbic connectivity has been linked to heightened anxiety and emotional dysregulation (6, 20). Despite these advances, significant limitations remain in the existing literature. First, considerable sample heterogeneity across studies—particularly the inclusion of medicated patients or those with prior treatment exposure—may confound the interpretation of findings and obscure the neural signatures specific to the disorder itself (3, 9, 21). Second, while seed-based ACC functional connectivity analyses have been reported in OCD, including in unmedicated or treatment-naïve samples (3, 7, 12, 22), methodological heterogeneity across studies — particularly differences in seed definition, sample characteristics, and analytic approaches — limits direct comparability and underscores the value of further well-controlled replication in treatment-naïve cohorts. Third, the relationship between functional connectivity abnormalities and clinical symptom severity has been inconsistently studied, with only a few studies reporting have found direct brain-behavior links (12, 23). These gaps underscore the need for well-controlled studies in treatment-naïve OCD patients to elucidate the ACC’s inherent functional structure and therapeutic significance.

To address the aforementioned limitations, the present study adopted a rigorous methodological approach by specifically recruiting treatment-naïve OCD patients who had never received pharmacological or psychotherapeutic interventions, thereby minimizing potential confounding effects of medication or prior treatment on brain functional connectivity patterns (3, 9). Furthermore, we selected a predefined anatomical mask of the anterior cingulate cortex (ACC) to serve as our primary seed-based region of interest (ROI) within a seed-driven whole-brain functional coupling assessment, allowing for a comprehensive whole-brain examination of ACC-centered network alterations that may have been overlooked in previous studies using independent component analysis or graph-theoretical approaches. This study’s two main goals were to: first, compare the intrinsic functional connectivity patterns of the ACC in untreated OCD patients to demographically matched healthy controls while they were at rest;and second, to examine the correlations between ACC connectivity abnormalities and clinical symptom severity as measured by standardized rating scales including HAMD, HAMA, and Y-BOCS.) By characterizing ACC functional connectivity alterations in a treatment-naïve sample, this study intends to shed more light on the underlying neuropathological causes of OCD, independent of medication-induced neuroplastic changes.

## 2 Materials and procedures

### 2.1 Participants

#### 2.1.1 Group of patients

Patients with neurosis and insomnia were collected from the outpatient clinics of Nanjing Medical University’s Affiliated Brain Hospital between January 2012 and October 2015.

In order to be eligible, (1) a person had to be between the ages of 18 and 50, right-handed, and have completed at least nine years of school; (2) fulfill the diagnostic criteria for OCD as outlined in the DSM-IV-TR; (3) not be on any centrally acting pharmacological agents, encompassing selective serotonin reuptake inhibitors (SSRIs); (4) a minimum aggregate severity score of 16 or above on the Y-BOCS (or subscale score ≥ 10 for patients with obsessions or compulsions only); (5) a total score of less than 24 on the HAMD-17.

The following were among the exclusion criteria: (1) comorbid diagnosis of other Axis I psychiatric conditions, including schizophrenia and mood disorders; (2) presence of other neurological diseases or history of any major physical illness; (3) a history of alcohol or substance abuse or dependency; (4) pregnancy or lactation; (5) previous surgical treatment, gamma knife radiosurgery, deep brain stimulation (DBS), or electroconvulsive therapy; and (6) contraindications for magnetic resonance imaging examination.

#### 2.1.2 Control group

Healthy volunteers were clinically recruited for the control group. Inclusion criteria were: (1) The person must be right-handed, between the ages of 18 and 50, and have completed at least nine years of education; (2) negative history of mental illness in two or three generations; (3) no history of major physical illness; (4) not have any medical conditions that would prevent them from participating in the study voluntarily and with written informed consent.

This investigation was approved by the Ethics Committee of the Affiliated Brain Hospital at Nanjing Medical University and conducted in accordance with the Declaration of Helsinki, and all participants — including patients and healthy controls — voluntarily enrolled with written informed consent.

### 2.2 Methods

#### 2.2.1 Clinical diagnosis and assessment

An associate chief psychiatrist used the DSM-IV-TR’s OCD diagnostic criteria (24) to diagnose enrolled individuals. Each participant was screened using the Structured Clinical Interview for DSM-IV Axis I Disorders, Non-patient Edition (SCID). Clinical interviews and psychological assessments were performed by two psychiatrists who had received standardized training in the use of rating scales and score consistency. The inter-rater reliability between the two evaluators was excellent, with intraclass correlation coefficients (ICCs; two-way random-effects model, absolute agreement; ICC (2, 1)) exceeding 0.85 for all rating scales. The assessment battery included the Y-BOCS, the HAMD-17, the HAMA, and a self-designed questionnaire.

The gold standard for determining the intensity of obsessive-compulsive symptoms is the semi-structured, clinician-administered Y-BOCS (25). It has ten items, five of which assess compulsions (repetitive actions) and five of which assess obsessions (intrusive thoughts). Each item is rated on a 5-point (0–4) scale, yielding a total score that ranges from 0 to 40. In Chinese populations, the Y-BOCS has shown outstanding psychometric qualities, including exceptional test-retest reliability and great internal consistency (Cronbach’s α > 0.80). The dimensional profile of obsessive-compulsive symptom burden was evaluated through the clinician-administered Yale-Brown Obsessive-Compulsive Scale Symptom Checklist (Y-BOCS-SC).

A popular clinician-rated scale for gauging the intensity of depression symptoms is the HAMD-17 (26). It consists of 17 measures that evaluate mood, guilt, suicidal thoughts, insomnia, psychomotor symptoms, and physical complaints, among other aspects of depression, with total scores ranging from 0 to 52. In Chinese psychiatric populations, the scale has demonstrated strong validity and reliability (27).

A standardized clinician-administered psychometric instrument Hamilton Anxiety Rating Scale (HAMA) was used by clinicians to systematically quantify the intensity of anxious symptomatology (28). It includes 14 questions that address psychological and physical symptoms of anxiety, such as tension, anxiety, sleep problems, autonomic symptoms, cognitive impairment, and anxious mood. The overall score range is 0–56, with each item receiving a score between 0 (not present) and 4 (extremely severe). The HAMA has been verified in Chinese populations with strong construct validity and satisfactory internal consistency (Cronbach’s α > 0.75) (27).

#### 2.2.2 MRI scanning

The Department of Radiology at our hospital used a Siemens 3.0-Tesla MRI system (Siemens Healthineers, Erlangen, Germany) paired with a 32-channel standard head coil to perform all MRI scans. On the scanning table, participants were laid supine with their heads securely fastened and comfortably positioned. To lessen the disturbance from scanner noise, earplugs were utilized. Participants were directed to keep motionless with eyes shut and maintain a wakeful state during scanning. All scanning procedures were conducted by one experienced radiologist proficient in MRI operations. All subjects underwent routine clinical sequencing employing fluid-attenuated inversion recovery (FLAIR) to rule out organic brain lesions.

Resting-state functional MRI BOLD signals were acquired using a gradient-echo echo-planar imaging (GRE-EPI) sequence with these specifications: matrix = 64 × 64, flip angle = 90°, slice thickness = 4 mm, gap = 0 mm, echo time (TE) = 25 ms, and repetition time (TR) = 2000 ms, and a total of N(240) volumes (acquisition time = [N × 2] s, i.e., 8 min). Whole-brain high-resolution three-dimensional T1-weighted magnetization-prepared structural images were acquired with the following settings: flip angle = 9°, TR = 1900 ms, TE = 2.48 ms, inversion time (TI) = 900 ms, 176 slices, matrix = 256 × 256, slice thickness = 1 mm.

#### 2.2.3 Information processing

Imaging data processing and analytical procedures were carried out on the MATLAB 2012b platform using the Data Processing Assistant for Resting-State fMRI (DPARSF, http://www.restfmri.net) — a software package built on Statistical Parametric Mapping 8 (SPM8, http://www.fil.ion.ucl.ac.uk/spm) — alongside the REST toolbox (Resting-State fMRI Data Analysis Toolkit; http://www.restfmri.net), which provides standard modules for resting-state fMRI preprocessing and connectivity analyses. The detailed processing workflow was implemented as follows: all raw DICOM data were transcoded into NIfTI file format; the first 10 volumes were omitted to mitigate biases arising from early signal instability and participant acclimatization within the magnetic resonance scanning environment; slice-timing correction was subsequently applied so as to attenuate temporal discrepancies associated with sequential slice acquisition, and head motion correction was performed to extract six motion parameters (translational and rotational displacements along the x, y, and z axes). Participants exhibiting frame-wise translational displacement exceeding 2 mm or rotational deviation surpassing 2° were excluded from subsequent analyses to minimize the impact of head motion artifacts induced during fMRI scanning. To remove confounding physiological noise and low-frequency signal drift from the preprocessed imaging data, temporal band-pass filtering spanning 0.01–0.08 Hz was subsequently applied. All imaging volumes underwent spatially normalization into the Montreal Neurological Institute (MNI) standard template via affine and nonlinear deformation procedures, followed by spatial resampling to an isotropic cubic voxel resolution of 3 × 3 × 3 mm³; spatial smoothing was concurrently performed by convolving the normalized volumes with a Gaussian kernel parameterized at a full width at half maximum (FWHM) of 6 mm. Nuisance covariates were subsequently removed through general linear model-based regression, including all six rigid-body head motion parameters alongside white matter signal, cerebrospinal fluid signal, and whole-brain mean signal time series. The bilateral anterior cingulate cortex was extracted via the Automated Anatomical Labeling (AAL) atlas-based parcellation framework to serve as a functionally defined seed region of interest (ROI). Subsequently, the ROI images corresponding to the bilateral anterior cingulate cortex underwent spatial resampling to conform to the uniform cubic voxel resolution of 3 × 3 × 3 mm³; the mean BOLD signal time series of this target region was then computed and correlated with every voxel across the entire brain to derive whole-brain resting-state functional connectivity maps. Finally, the resulting Pearson correlation coefficients underwent transformation to normally distributed z-scores via the Fisher r-to-z normalization procedure, ensuring the data conformed to a normal distribution.

### 2.3 Statistical analysis

SPSS 20.0 was used to evaluate clinical and demographic data. Independent samples t-tests were employed to compare continuous variables across different groups, whereas chi-square tests were used to analyze categorical variables.

The REST software package on the MATLAB platform was used to analyze imaging data. Bilateral anterior cingulate cortex served as the seed of interest for a hypothesis-driven seed-based whole-brain functional connectivity study. Cluster-level significance criterion was set at a family-wise error-corrected threshold of P < 0.05 (which corresponds to a voxel-level uncorrected threshold of P < 0.01, with minimum cluster extent threshold based on AlphaSim simulation); the statistical data were adjusted for multiple comparisons using AlphaSim correction.

The peak coordinates of brain areas exhibiting aberrant functional connectivity were used to center spherical regions of interest with a 5 mm radius for correlation studies. Mean functional connectivity values were extracted from these ROIs and subjected to Pearson (two-tailed) correlation analysis with clinical scale scores (Y-BOCS, HAMD-17, and HAMA). Subsequently, partial correlation analysis was performed controlling for sex, age, and years of schooling.

## 3 Results

### 3.1 Comparison of demographic and clinical characteristics

This study recruited 46 OCD patients and 33 healthy control participants. No statistically significant differences were observed between the two groups in terms of age (28.63 ± 6.90 years vs. 25.61 ± 7.43 years, P = 0.067), years of schooling (15.17 ± 2.132 vs. 14.42 ± 1.985, P = 0.117), or sex distribution (male/female: 24/22 vs. 17/16, P = 0.954). In contrast, the OCD patient group exhibited significantly higher scores than the control group on the Y-BOCS, obsession subscale, compulsion subscale, HAMD-17, and HAMA, with all comparisons yielding P < 0.001 (**Table 1**).

**Table 1:** Comparison of demographic and clinical characteristics between patient and control groups.

Variables	OCD Patients (n=46)	Healthy Controls (n=33)	P value
Age (years)	28.63 ± 6.89	25.61 ± 7.43	0.067
Gender (Male/Female)	24/22	17/16	0.954
Education (years)	15.17 ± 2.13	14.42 ± 1.99	0.117
Y-BOCS	24.67 ± 5.09	0.64 ± 1.14	<0.001*
YBOCs-obsessions	12.33 ± 2.59	0.42 ± 0.71	<0.001*
YBOCs-compulsions	12.35 ± 2.60	0.21 ± 0.60	<0.001*
HAMD-17	11.89 ± 4.79	1.70 ± 2.07	<0.001*
HAMA	14.93 ± 6.35	1.27 ± 1.55	<0.001*

Y-BOCS, Yale-Brown Obsessive Compulsive Scale; HAMD-17, Hamilton Depression Rating Scale-17; HAMA, Hamilton Anxiety Rating Scale.

*P < 0.05 indicates statistical significance.

### 3.2 Analysis of the bilateral anterior cingulate cortex’s functional connectivity in healthy controls and drug-naive OCD patients

**Figure 1:**
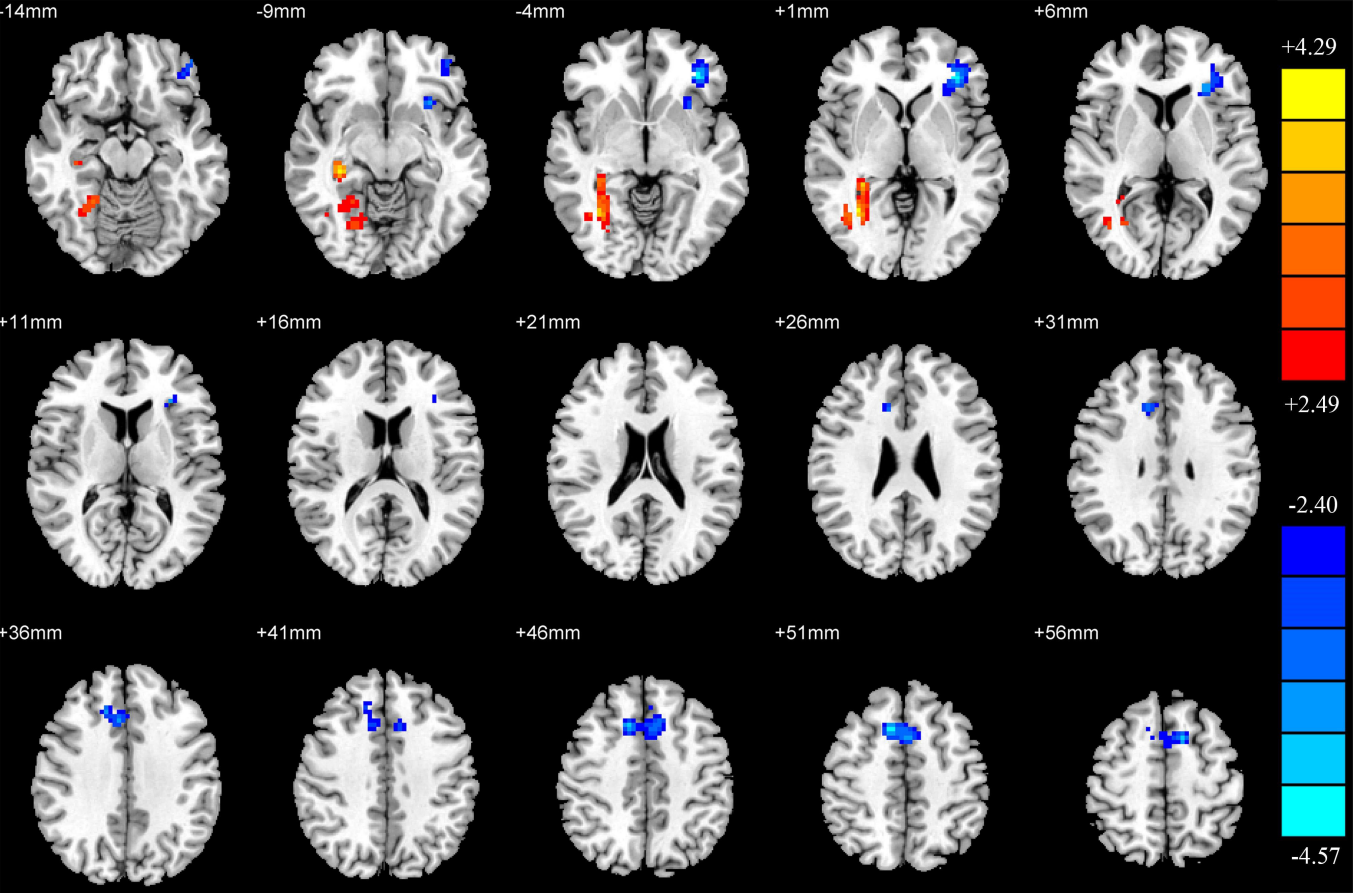
Altered ACC functional connectivity in OCD patients. Brain regions showing significantly altered resting-state functional connectivity with the anterior cingulate cortex (ACC) in treatment-naïve OCD patients compared to healthy controls (AlphaSim-corrected P < 0.05), displayed on axial slices of the MNI standard brain from −14 mm to +56 mm along the z-axis. Warm colors (red–yellow) indicate increased connectivity (right insula, right hippocampus, right fusiform gyrus); cool colors (blue–cyan) indicate decreased connectivity (left inferior frontal gyrus, right supplementary motor area). The color bar represents T-values (−4.57 to +4.29). Peak coordinates and cluster details are provided in **Table 2**.

Seed-based whole-brain functional connectivity analysis revealed significant differences in anterior cingulate cortex connectivity between the OCD patient group and healthy control participants. Specifically, the OCD patient cohort displayed reduced functional connectivity with the right supplementary motor area (BA 32, peak MNI coordinates: 12, 15, 48; T = -4.44; cluster size: 113 voxels) and the left inferior frontal gyrus (BA 47, peak MNI coordinates: -36, 37, 0; T = -4.34; cluster size: 116 voxels). Conversely, the patient group showed increased functional connectivity with the right insula (BA 48, peak MNI coordinates: 29, 18, -6; T = 3.63; cluster size: 77 voxels), the right hippocampus (BA 20, peak MNI coordinates: 33, -30, -9; T = 4.27; cluster size: 84 voxels), and the right fusiform gyrus (BA 37, peak MNI coordinates: 32, -56, -5; T = 3.21; cluster size: 100 voxels). All findings were adjusted for multiple comparisons using AlphaSim correction, with a statistical significance threshold set at P < 0.05. Further details are provided in **Figure 1** and **Table 2**.

**Table 2:** Peak coordinates of brain regions with abnormal functional connectivity of the anterior cingulate cortex in patient group.

Brain Region	Hemisphere	BA	MNI Coordinates			T value	K value
			x	y	z		
Decreased							
Inferior Frontal Gyrus	L	47	-36	37	0	-4.34	116
Supplementary Motor Area	R	32	12	15	48	-4.44	113
Increased							
Insula	R	48	29	18	-6	3.63	77
Hippocampus	R	20	33	-30	-9	4.27	84
Fusiform Gyrus	R	37	32	-56	-5	3.21	100

BA, Brodmann area; MNI, Montreal Neurological Institute; x, y, z, MNI coordinate axes (mm); L, Left; R, Right; AlphaSim corrected, P < 0.05 for statistical significance; K value, cluster size (number of voxels).

### 3.3 Correlation analysis between brain regions with abnormal anterior cingulate cortex functional connectivity and clinical characteristics

The peak coordinates of the five brain areas exhibiting aberrant functional connectivity were used to form spherical regions of interest with a radius of 5 mm. Mean functional connectivity values were extracted from these ROIs and subjected to Pearson correlation analysis with Y-BOCS, HAMD-17, and HAMA scores. Our findings revealed a positive correlation between the total Y-BOCS score and the functional connectivity of the left inferior frontal gyrus with the anterior cingulate cortex (r = 0.351, P = 0.017). No statistically discernible functional connectivity associations were detected between the other brain areas (right supplementary motor area, right insula, right hippocampus, right fusiform gyrus) and Y-BOCS scores (P > 0.05). Furthermore, no significant relationships between any brain region and HAMA or HAMD-17 scores were discovered (P > 0.05). Statistically non-significant correlations were identified among the remaining brain regions and clinical rating scale scores (Y-BOCS, HAMD-17, and HAMA); however, when covariate-controlled partial correlation analysis was performed while statistically adjusting for sex, age, and formal educational attainment as sociodemographic covariates, the resting-state functional connectivity strength of the coupling between the left inferior frontal gyrus and anterior cingulate cortex remained significantly positively associated with total Y-BOCS scores (r = 0.351, P = 0.017).

## 4 Discussion

This study examined the anterior cingulate cortex’s intrinsic functional connectivity patterns in patients with OCD who had not yet received therapy while at rest. Our primary findings revealed a specific pattern of ACC connectivity alterations characterized by both hypoconnectivity and hyperconnectivity with distinct brain regions. Compared with healthy control participants, drug-naïve OCD patients showed significantly reduced functional connectivity between the anterior cingulate cortex (ACC) and both the left inferior frontal gyrus (BA 47) and right supplementary motor area (BA 32). Conversely, these patients displayed higher functional connectivity between the ACC and the right hippocampus (BA 20), right insula (BA 48), and right fusiform gyrus (BA 37). The functional connectivity strength between the left inferior frontal gyrus and ACC was strongly positively associated with Y-BOCS total scores, suggesting a clear relationship between the severity of symptoms and this connectivity abnormality. Clinical indicators such as HAMD-17 or HAMA scores did not significantly correlate with ACC connection with other brain areas. These medication-naïve findings demonstrate that ACC connectivity abnormalities are intrinsic to OCD rather than medication-related, and reveal the ACC’s role in integrating distributed cognitive, emotional, and sensory networks in this disorder (29, 30).

The ACC’s functional connection with two important brain regions—the left inferior frontal gyrus and the right supplementary motor area—was shown to be severely reduced. The left inferior frontal gyrus, specifically BA 47, is essential for cognitive control, response inhibition, and cognitive flexibility. Convergent evidence has implicated the IFG in error monitoring and inhibitory control processes essential for suppressing unwanted thoughts and behaviors (31). Notably, the significant positive correlation between left IFG-ACC connectivity strength and Y-BOCS total scores in this study suggests that greater disruption of this circuit is associated with more severe obsessive-compulsive symptoms (). The absence of significant correlations with HAMD-17 and HAMA scores is likely attributable to the restricted score range imposed by our inclusion criteria (HAMD-17 < 24; mean = 11.89 ± 4.79), which reduced statistical power to detect affective brain-behavior associations; moreover, the mild-to-moderate depressive and anxiety symptoms observed in our sample likely reflect reactive distress secondary to OCD symptom burden rather than independent affective pathology — consistent with our explicit exclusion of comorbid Axis I mood and anxiety disorders — and may thus be more closely linked to the observed limbic hyperconnectivity (ACC–hippocampus and ACC–insula) than to the ACC–IFG cognitive control pathway. The present finding converges with earlier neuroimaging evidence demonstrating that poorer motor response inhibition in OCD was linked to aberrantly elevated resting-state functional connectivity between the pre-supplementary motor region and the IFG, and meaningfully extends the existing mechanistic understanding to the ACC-IFG pathway. The ACC-IFG circuit may serve as a crucial neural substrate for cognitive control deficits in OCD, whereby diminished connectivity compromises the ability to inhibit intrusive obsessive thoughts and compulsive urges. Similarly, the right supplementary motor area (BA 32) is integral to motor planning, voluntary motor control, and behavioral inhibition (3, 32). The observed reduction in ACC-SMA connectivity may contribute to the repetitive, ritualistic motor behaviors characteristic of OCD, as the SMA is critically involved in the initiation and sequencing of complex motor acts. Previous studies have reported abnormal SMA activation during symptom provocation and motor inhibition tasks in OCD patients (18), supporting the notion that disrupted ACC-SMA functional coupling underlies the loss of voluntary control over compulsive behaviors. Nonetheless, the literature contains contradictory results. In contrast to our result of decreased ACC-SMA connection, Tomiyama et al. (2022) observed increased functional connectivity between pre-SMA and IFG in OCD patients (). These discrepancies may reflect methodological differences in seed region selection (pre-SMA vs. ACC), sample characteristics (medication status), or the specific nodes examined within overlapping networks, underscoring the complex and potentially heterogeneous nature of frontal-motor circuit dysfunction in OCD.

Only the left IFG-ACC functional connectivity strength demonstrated a statistically robust positive neural-behavioral association with aggregate Y-BOCS obsessive-compulsive symptom severity composite scores, whereas no statistically meaningful neural-behavioral associations were detected between overall severity across symptoms or comorbid anxiety/depressive symptoms and the other abnormal connectivity patterns (right SMA, right insula, right hippocampus, and right fusiform gyrus). This is a significant clinical finding of the current study. This selective brain-behavior relationship suggests that ACC-IFG connectivity may represent a core neural substrate specifically linked to OCD symptom burden, potentially reflecting the integrity of top-down cognitive control mechanisms essential for suppressing obsessive-compulsive symptoms (12). The absence of correlations with other connectivity abnormalities may reflect methodological factors such as sample heterogeneity in symptom dimensions (14), or suggest that certain connectivity alterations represent trait markers rather than state-dependent features directly proportional to current symptom severity (33). Notably, disrupted ACC-IFG connectivity, given its significant association with symptom severity and the precedent of frontocortical connectivity as a predictor of neuromodulation outcomes, warrants further investigation as a potential biomarker for treatment stratification in OCD, though prospective longitudinal studies are needed to confirm its predictive validity (34). Moreover, these findings have implications for neuromodulation interventions: the ACC and IFG, as critical nodes showing symptom-related connectivity dysfunction, may serve as optimal targets for treatment-refractory OCD that may benefit from DBS or rTMS (35, 36).

There are a few limits to take into account. Firstly, we are unable to draw conclusions about the causal relationship between ACC connection anomalies and fundamental pathophysiological mechanisms or secondary effects of OCD due to the cross-sectional methodology. Second, the relatively modest sample size (46 OCD patients, 33 controls) may limit statistical power to detect subtle connectivity alterations and brain-behavior relationships, necessitating replication in larger cohorts. Third, we employed the bilateral ACC as a single, anatomically defined seed region without subdividing it into functionally distinct dorsal and ventral (subgenual) subregions. The dorsal ACC (dACC, BA 24/32) is predominantly engaged in cognitive control, conflict monitoring, and error detection, while the subgenual ACC (sgACC, BA 25) is more closely associated with affective regulation and mood homeostasis. This lack of subregional parcellation may have limited our ability to dissociate connectivity patterns specifically related to cognitive versus affective symptom dimensions. Future studies should adopt subregion-specific ACC seed analyses to more precisely characterize differential contributions of dACC and sgACC connectivity to OCD’s cognitive and affective symptom profiles. Fourth, we employed bilateral ACC as a single seed region without subdividing it into dorsal and ventral subregions, which have distinct functional profiles and differential involvement in cognitive versus affective processing. Fifth, the absence of longitudinal follow-up data prevents evaluation of whether baseline ACC connectivity abnormalities predict treatment response or clinical trajectory. Sixth, the healthy control group required a negative psychiatric family history across two to three generations. Although intended to minimize genetic confounding, this stringent criterion may have produced a “super-control” sample unrepresentative of the general population, potentially inflating observed group differences. Finally, OCD is characterized by symptom heterogeneity across multiple dimensions (contamination/washing, checking, symmetry/ordering, hoarding), yet we did not conduct symptom dimension-specific analyses, which may have obscured subtype-specific neural signatures.

In treatment-naïve OCD patients, this study found distinct patterns of ACC functional connectivity changes, including increased connectivity with the right insula, hippocampus, and fusiform gyrus and decreased connectivity with the right supplementary motor area and left inferior frontal gyrus. The significant correlation between left IFG-ACC connectivity strength and symptom severity underscores its clinical relevance as a potential neurobiological marker. These findings provide compelling evidence that intrinsic ACC connectivity disruptions, particularly within cognitive control networks, represent core pathophysiological features of OCD independent of medication effects. Our results advance understanding of OCD neurocircuitry and may inform precision medicine approaches, including patient stratification and targeted neuromodulation strategies for treatment-refractory cases.

## Data Availability

The raw data supporting the conclusions of this article will be made available by the authors, without undue reservation.

## References

[1] Hou JM Zhao M Zhang W Song LH Wu WJ Wang J . Resting-state functional connectivity abnormalities in patients with obsessive-compulsive disorder and their healthy first-degree relatives. J Psychiatry Neurosci. (2014) 39:304–11. doi: 10.1503/jpn.130220. PMID: 24866415 PMC4160359

[2] Anticevic A Hu S Zhang S Savic A Savic A Billingslea E . Global resting-state functional magnetic resonance imaging analysis identifies frontal cortex, striatal, and cerebellar dysconnectivity in obsessive-compulsive disorder. Biol Psychiatry. (2014) 75:595–605. doi: 10.1016/j.biopsych.2013.10.021. PMID: 24314349 PMC3969771

[3] Cheng Y Xu J Nie B Luo C Yang T Li H . Abnormal resting-state activities and functional connectivities of the anterior and the posterior cortexes in medication-naïve patients with obsessive-compulsive disorder. PloS One. (2013) 8:e67478. doi: 10.1371/journal.pone.0067478. PMID: 23840714 PMC3696097

[4] Calzà J Gürsel DA Schmitz-Koep B Bremer B Reinholz L Berberich G . Altered cortico-striatal functional connectivity during resting state in obsessive-compulsive disorder. Front Psychiatry. (2019) 10:319. doi: 10.3389/fpsyt.2019.00319. PMID: 31133898 PMC6524661

[5] Vaghi MM Vértes PE Kitzbichler MG Apergis-Schoute AM van der Flier FE Fineberg NA . Specific frontostriatal circuits for impaired cognitive flexibility and goal-directed planning in obsessive-compulsive disorder: Evidence from resting-state functional connectivity. Biol Psychiatry. (2017) 81:708–17. doi: 10.1016/j.biopsych.2016.08.009. PMID: 27769568 PMC6020061

[6] Jung WH Kang DH Kim E Shin KS Jang JH Kwon JS . Abnormal corticostriatal-limbic functional connectivity in obsessive-compulsive disorder during reward processing and resting-state. NeuroImage Clin. (2013) 3:27–38. doi: 10.1016/j.nicl.2013.06.013. PMID: 24179846 PMC3791288

[7] Yun JY Jang JH Jung WH Shin NY Kim SN Hwang JY . Executive dysfunction in obsessive-compulsive disorder and anterior cingulate-based resting state functional connectivity. Psychiatry Investig. (2017) 14:333–43. doi: 10.4306/pi.2017.14.3.333. PMID: 28539952 PMC5440436

[8] Zhu Y Fan Q Zhang H Qiu J Tan L Xiao Z . Altered intrinsic insular activity predicts symptom severity in unmedicated obsessive-compulsive disorder patients: a resting state functional magnetic resonance imaging study. BMC Psychiatry. (2016) 16:104. doi: 10.1186/s12888-016-0806-9. PMID: 27084762 PMC4833895

[9] Yang X Hu X Tang W Li B Yang Y Gong Q . Intrinsic brain abnormalities in drug-naive patients with obsessive-compulsive disorder: A resting-state functional MRI study. J Affect Disord. (2019) 245:861–8. doi: 10.1016/j.jad.2018.11.080. PMID: 30699871

[10] Hou J Wu W Lin Y Wang J Zhou D Guo J . Localization of cerebral functional deficits in patients with obsessive-compulsive disorder: a resting-state fMRI study. J Affect Disord. (2012) 138:313–21. doi: 10.1016/j.jad.2012.01.022. PMID: 22331021

[11] Xu Y Han S Wei Y Zheng R Cheng J Zhang Y . Abnormal resting-state effective connectivity in large-scale networks among obsessive-compulsive disorder. Psychol Med. (2024) 54:350–8. doi: 10.1017/S0033291723001228. PMID: 37310178

[12] Tian L Meng C Jiang Y Tang Q Wang S Xie X . Abnormal functional connectivity of brain network hubs associated with symptom severity in treatment-naive patients with obsessive-compulsive disorder: A resting-state functional MRI study. Prog Neuro-Psychopharmacol Biol Psychiatry. (2016) 66:104–11. doi: 10.1016/j.pnpbp.2015.12.003. PMID: 26683173

[13] Stern ER Fitzgerald KD Welsh RC Abelson JL Taylor SF . Resting-state functional connectivity between fronto-parietal and default mode networks in obsessive-compulsive disorder. PloS One. (2012) 7:e36356. doi: 10.1371/journal.pone.0036356. PMID: 22570705 PMC3343054

[14] Liu J Cao L Li H Gao Y Bu X Liang K . Abnormal resting-state functional connectivity in patients with obsessive-compulsive disorder: A systematic review and meta-analysis. Neurosci Biobehav Rev. (2022) 135:104574. doi: 10.1016/j.neubiorev.2022.104574. PMID: 35151769

[15] Biswal BB Mennes M Zuo XN Gohel S Kelly C Smith SM . Toward discovery science of human brain function. Proc Natl Acad Sci USA. (2010) 107:4734–9. doi: 10.1073/pnas.0911855107. PMID: 20176931 PMC2842060

[16] Beucke JC Sepulcre J Talukdar T Linnman C Zschenderlein K Endrass T . Abnormally high degree connectivity of the orbitofrontal cortex in obsessive-compulsive disorder. JAMA Psychiatry. (2013) 70:619–29. doi: 10.1001/jamapsychiatry.2013.173. PMID: 23740050

[17] Göttlich M Krämer UM Kordon A Hohagen F Zurowski B . Decreased limbic and increased fronto-parietal connectivity in unmedicated patients with obsessive-compulsive disorder. Hum Brain Mapp. (2014) 35:5617–32. doi: 10.1002/hbm.22574. PMID: 25044747 PMC6868939

[18] Fitzgerald KD Stern ER Angstadt M Nicholson-Muth KC Maynor MR Welsh RC . Altered function and connectivity of the medial frontal cortex in pediatric obsessive-compulsive disorder. Biol Psychiatry. (2010) 68:1039–47. doi: 10.1016/j.biopsych.2010.08.018. PMID: 20947065 PMC2988474

[19] Fan Q Yan X Wang J Chen Y Wang X Li C . Abnormalities of white matter microstructure in unmedicated obsessive-compulsive disorder and changes after medication. PloS One. (2012) 7:e35889. doi: 10.1371/journal.pone.0035889. PMID: 22558258 PMC3338776

[20] Eng GK Sim K Chen SH . Meta-analytic investigations of structural grey matter, executive domain-related functional activations, and white matter diffusivity in obsessive compulsive disorder: an integrative review. Neurosci Biobehav Rev. (2015) 52:233–57. doi: 10.1016/j.neubiorev.2015.03.002. PMID: 25766413

[21] Peng Z Lui SS Cheung EF Jin Z Miao G Jing J . Brain structural abnormalities in obsessive-compulsive disorder: converging evidence from white matter and grey matter. Asian J Psychiatr. (2012) 5:290–6. doi: 10.1016/j.ajp.2012.07.004. PMID: 23174435

[22] Chen Y Juhás M Greenshaw AJ Hu Q Meng X Cui H . Abnormal resting-state functional connectivity of the left caudate nucleus in obsessive-compulsive disorder. Neurosci Lett. (2016) 623:57–62. doi: 10.1016/j.neulet.2016.04.030. PMID: 27143323

[23] Gürsel DA Avram M Sorg C Brandl F Koch K . Frontoparietal areas link impairments of large-scale intrinsic brain networks with aberrant fronto-striatal interactions in OCD: a meta-analysis of resting-state functional connectivity. Neurosci Biobehav Rev. (2018) 87:151–60. doi: 10.1016/j.neubiorev.2018.01.016. PMID: 29410103

[24] Zimmerman M Jampala VC Sierles FS Taylor MA . DSM-IV: a nosology sold before its time? Am J Psychiatry. (1991) 148:463–7. doi: 10.1176/ajp.148.4.463 2006692

[25] Melli G Avallone E Moulding R Pinto A Micheli E Carraresi C . Validation of the Italian version of the Yale-Brown Obsessive Compulsive Scale-Second Edition (Y-BOCS-II) in a clinical sample. Compr Psychiatry. (2015) 60:86–92. doi: 10.1016/j.comppsych.2015.03.005. PMID: 25842194

[26] Li L Wan A Zhao M Yu L Han Y Mi K . Effect of Shengyang Yiwei Decoction combined with selective serotonin reuptake inhibitor antidepressants on HAMD-17 score and somatic symptoms in patients with depression. Pak J Med Sci. (2025) 41:205–9. doi: 10.12669/pjms.41.1.9835. PMID: 39867783 PMC11755288

[27] Liu J Jia F Li C Yuan H Yang H Yang R . Association between body mass index and suicide attempts in Chinese patients of a hospital in Shanxi district with first-episode drug-naïve major depressive disorder. J Affect Disord. (2023) 339:377–83. doi: 10.1016/j.jad.2023.06.064. PMID: 37393956

[28] Hamilton M . The assessment of anxiety states by rating. Br J Med Psychol. (1959) 32:50–5. doi: 10.1111/j.2044-8341.1959.tb00467.x. PMID: 13638508

[29] Tomiyama H Murayama K Nemoto K Tomita M Hasuzawa S Mizobe T . Increased functional connectivity between presupplementary motor area and inferior frontal gyrus associated with the ability of motor response inhibition in obsessive-compulsive disorder. Hum Brain Mapp. (2022) 43:974–84. doi: 10.1002/hbm.25699. PMID: 34816523 PMC8764470

[30] Fan J Xia J Liu Q Wang X Du H Gao F . Neural substrates for dissociation of cognition inhibition in autogenous- and reactive-type obsessive-compulsive disorder. J Psychiatr Res. (2023) 165:150–7. doi: 10.1016/j.jpsychires.2023.07.031. PMID: 37499486

[31] Zhong Z Ou Y Chen Y Li P Shi H Lv D . Reduced functional connectivity of the right dorsolateral prefrontal cortex at rest in obsessive-compulsive disorder. Brain Behav. (2024) 14:e3333. doi: 10.1002/brb3.3333. PMID: 38376021 PMC10784187

[32] Kang DH Jang JH Han JY Kim JH Jung WH Choi JS . Neural correlates of altered response inhibition and dysfunctional connectivity at rest in obsessive-compulsive disorder. Prog Neuro-Psychopharmacol Biol Psychiatry. (2013) 40:340–6. doi: 10.1016/j.pnpbp.2012.11.001. PMID: 23146681

[33] Chen Y Ou Y Lv D Ma J Zhan C Yang R . Decreased nucleus accumbens connectivity at rest in medication-free patients with obsessive-compulsive disorder. Neural Plast. (2021) 2021:9966378. doi: 10.1155/2021/9966378. PMID: 34158811 PMC8187042

[34] Chen X Wang Z Lv Q Luo C Yang T Li H . Common and differential connectivity profiles of deep brain stimulation and capsulotomy in refractory obsessive-compulsive disorder. Mol Psychiatry. (2022) 27:1020–30. doi: 10.1038/s41380-021-01358-w. PMID: 34703025

[35] Ji GJ Xie W Yang T Wu Q Sui P Bai T . Pre-supplementary motor network connectivity and clinical outcome of magnetic stimulation in obsessive-compulsive disorder. Hum Brain Mapp. (2021) 42:3833–44. doi: 10.1002/hbm.25468. PMID: 34050701 PMC8288080

[36] Yin D Zhang C Lv Q Chen X Gong H Zhan S . Dissociable frontostriatal connectivity: Mechanism and predictor of the clinical efficacy of capsulotomy in obsessive-compulsive disorder. Biol Psychiatry. (2018) 84:926–36. doi: 10.1016/j.biopsych.2018.04.006. PMID: 29778276

